# Synthesis of Ag_2_O/Ag Nanoparticles Using Puerarin: Characterization, Cytotoxicity, *In Ovo* Safety Profile, Antioxidant, and Antimicrobial Potential Against Nosocomial Pathogens

**DOI:** 10.3390/jfb16070258

**Published:** 2025-07-11

**Authors:** Sergio Liga, Raluca Vodă, Lavinia Lupa, Elena-Alina Moacă, Delia Muntean, Lucian Barbu-Tudoran, Maria Suciu, Vlad Socoliuc, Francisc Péter

**Affiliations:** 1 Faculty of Chemical Engineering, Biotechnologies and Environmental Protection, Politehnica University Timișoara, Vasile Pârvan No. 6, 300223 Timișoara, Romania; sergio.liga96@gmail.com (S.L.); lavinia.lupa@upt.ro (L.L.); francisc.peter@upt.ro (F.P.); 2 University Clinic of Toxicology, Drug Industry, Management and Legislation, Faculty of Pharmacy, “Victor Babeș” University of Medicine and Pharmacy Timișoara, Eftimie Murgu Square No. 2, 300041 Timișoara, Romania; alina.moaca@umft.ro; 3 Research Center for Pharmaco-Toxicological Evaluation, “Victor Babeș” University of Medicine and Pharmacy Timișoara, Eftimie Murgu Square No. 2, 300041 Timișoara, Romania; 4 Multidisciplinary Research Center on Antimicrobial Resistance, Department of Microbiology, Faculty of Medicine, “Victor Babeș” University of Medicine and Pharmacy Timișoara, Eftimie Murgu Square No. 2, 300041 Timișoara, Romania; muntean.delia@umft.ro; 5 Electron Microscopy Laboratory “Prof. C. Craciun”, Faculty of Biology & Geology, “Babes-Bolyai” University, 5-7 Clinicilor Street, 400006 Cluj-Napoca, Romania; lucian.barbu@ubbcluj.ro; 6 Electron Microscopy Integrated Laboratory, National Institute for R&D of Isotopic and Molecular Technologies, 67-103 Donat Street, 400293 Cluj-Napoca, Romania; 7 Romanian Academy–Timişoara Branch, Center of Fundamental and Advanced Technical Research, Laboratory of Magnetic Fluids, 24 Mihai Viteazul Avenue, 300223 Timişoara, Romania; vsocoliuc@gmail.com; 8 Renewable Energy Research Institute-ICER, Politehnica University Timișoara, Gavril Musicescu Street No. 138, 300501 Timișoara, Romania

**Keywords:** silver oxide, Puerarin, green synthesis, cytotoxicity, HET-CAM assay, antibacterial agent

## Abstract

**(1) Background**: Our study investigates the green synthesis of Ag_2_O/Ag nanoparticles using the isoflavone Puerarin as a bioreductor. **(2) Methods**: The PUE@Ag_2_O/Ag nanoparticles were characterized using various techniques, including X-ray diffraction (XRD), Fourier-transform infrared spectroscopy (FTIR), electronic microscopy (TEM, SEM), energy dispersive X-ray spectroscopy (EDX), and dynamic light scattering (DLS). Biological activities were assessed through antimicrobial testing, cytotoxicity assays on human keratinocytes and melanoma cells, and an in ovo screening using the HET-CAM assay. **(3) Results**: The formation of crystalline Ag_2_O/Ag nanoparticles with sizes below 100 nm was accomplished with Puerarin. Despite their high cytotoxicity at all tested concentrations, the nanoparticles showed antioxidant activity with IC_50_ 981.5 ± 94.2 μg/mL, antibacterial activity against several clinically relevant nosocomial strains (*Streptococcus pyogenes*, *Staphylococcus aureus*, *Escherichia coli*, *Pseudomonas aeruginosa*), and no local irritant effects or inhibition of angiogenesis in the HET-CAM assay. **(4) Conclusions**: This study provides insights into the synthesis, characterization, and biological profile of PUE@Ag_2_O/Ag nanoparticles for potential biomedical applications.

## 1. Introduction

Healthcare-associated infections (HAIs) are infections acquired during healthcare delivery and were not present or incubated at the time of patient admission [1]. Hospitals, long-term care facilities, and outpatient clinics are among the healthcare settings where these infections can occur and develop after discharge [2]. The microbial etiology of HAIs is diverse, with pathogenic strains such as Gram-positive pathogens (e.g., *Staphylococcus aureus*, methicillin-resistant (MRSA), and *Enterococcus* spp.) frequently involved in bloodstream and surgical site infections [1]. Among Gram-negative bacteria (e.g., *Escherichia coli*, *Klebsiella pneumoniae*, *Pseudomonas aeruginosa*, *Enterobacter* spp., *Acinetobacter baumannii*) that are commonly implicated, especially in urinary tract infections, pneumonia, and device-associated infections [1,3]. The presence of multidrug resistance (MDR) in many of these pathogenic strains can complicate treatment and increase the risk of adverse outcomes [3].

According to an assessment of the latest scientific trends, phytocompounds extracted from medicinal plants are essential resources for the development of new medicines, particularly anti-cancer drugs [4,5]. Their therapeutic effectiveness is often impaired by their poor solubility, low stability, and limited bioavailability. Recent advancements in nanotechnology—particularly the synthesis of metallic nanoparticles—offer innovative strategies to overcome these limitations [6,7,8]. Silver oxide (Ag_2_O) nanoparticles have attracted significant attention in recent years due to their unique physicochemical properties and diverse applications across various fields, including catalysis, environmental remediation, sensors and biosensors, optoelectronic devices, and biomedical applications [9,10]. In addition, the use of silver nanoparticles as antimicrobial agents has been significant in healthcare-associated infections (HAIs), specifically *Staphylococcus aureus*, *Pseudomonas aeruginosa*, and *Escherichia coli* [11,12,13]. Multiple mechanisms are involved in their antibacterial effects, which include membrane disruption, oxidative stress caused by ROS, and interference with DNA replication and protein synthesis [11,14]. Integrating silver nanoparticles into various biomaterials, coatings, wound dressings, and medical tools presents a promising strategy for preventing HAI, but more evaluation is needed to assess their safety and resistance potential [14,15].

The synthesis of Ag_2_O nanoparticles can be achieved through various methods, including chemical reduction, electrochemical precipitation, sol–gel processes, and increasingly popular green synthesis techniques utilizing plant extracts, natural bioactive molecules, and microbial agents [9,15,16]. These methods provide a cost-effective and environmentally friendly approach to silver oxide nanoparticle production and enhance the stability and functionality of the nanoparticles produced [16]. Sustainable synthesis, also called green synthesis, is a viable alternative to conventional methods by using plant extracts, microorganisms, or biomolecules as capping and reducing agents for nanoparticle fabrication [17]. Varieties of polyphenolic compounds, including flavonoids, which are secondary metabolites of plants, have emerged as efficient natural reducing and stabilizing agents for the green synthesis of Ag_2_O/Ag nanoparticles [18,19,20]. The green synthesis of Ag_2_O/Ag NPs not only leverages phytocompounds as reducing and stabilizing agents but also enhances the biological functionality of the resulting nanomaterials [21,22,23,24,25]. This synergistic integration of phytochemicals and silver oxide nanostructures presents a powerful platform for developing novel therapeutic agents with improved efficacy and safety profiles. Both Ag and Ag_2_O NPs are amazing nanomaterials with tremendous application possibilities, as it was highlighted in several recent reviews [26,27,28]. However, while the chemical synthesis of either Ag or Ag_2_O NPs from AgNO_3_ can be easily accomplished by selecting the appropriate reagents and conditions (e.g., NaBH_4_ for Ag, or NaOH for Ag_2_O), in the case of the so-called “green synthesis”—using plant extracts or specific components obtained from plants—the nature of the formed compound is not obvious. Although these natural extracts or compounds are considered reducing agents and should lead to the reduction of silver ions into Ag, the mechanism, hypothesized as a phytochemically assisted reduction [29], is more complex. According to several reports, the possible mechanism could also imply the initial formation of AgOH due to the interaction with the phenolic hydroxyl groups (-OH) of the phytochemicals, followed by its conversion into Ag_2_O [30,31]. Therefore, some reports labeled them as silver/silver oxide heterostructures [32,33], and we adopted the same terminology, also considering the possibility of their interconversion in aqueous solutions [34].

Most of the green synthesis methods of Ag_2_O/Ag NPs reported in the literature use plant extracts as reducing and endcapping agents, as also specified in several recent reviews, such as those of Vidyasagar et al. [27] and Dhaka et al. [28]. These extracts contain, alongside flavonoids, several other biomolecules, which can be useful in the biosynthesis process, like various other polyphenolic compounds, carbohydrates, sapogenins, terpenoids, alkaloids, etc., having the advantage of the relatively low cost of the crude extract. The major disadvantage of the plant extracts is obviously the variation in the chemical composition, even if the same extraction method is used [35]. The risk assessment and limitations of silver nanoparticles synthesized using plant extract were pointed out in an excellent review by Velidandi et al. [36]. Until now, an efficient, viable, and environmentally friendly green route to obtain Ag_2_O/Ag NPs using the natural reducing capacity of plant extracts has not yet been validated. Therefore, identification, separation, and utilization of certain biomolecules present in plants that can mediate the Ag_2_O/Ag NP production still represent an important research target. In this respect, the utilization of flavonoids, which are already used in nutraceuticals and medicines, having already established extraction and purification procedures, looks attractive. In fact, such studies were reported for several flavonoids such as apigenin [37], myricetin [38], hesperidin, naringin, and diosmin [19], or quercetin [23].

Puerarin (PUE) is a natural isoflavone extracted from the dried roots of the *Pueraria* genus plant, which has a variety of pharmacological effects [39,40]. In the biopharmaceutical classification system, Puerarin is classified as a class IV compound due to its low water solubility, lipid stability, and limited oral bioavailability [41]. Puerarin was demonstrated to possess various pharmacological activities, including cardioprotection, vasodilation, anti-inflammation, antioxidant, attenuating insulin resistance, and neuroprotection [42]. Although Puerarin has been identified as a promising flavonoid with significant biomedical potential, its role in the green synthesis of metal/metal oxide nanoparticle bioconjugates has barely been investigated [43,44]. Comprehensive studies are required to assess its effectiveness and underlying mechanisms as a biogenic reducing and capping agent within sustainable nanomaterial production frameworks. Moreover, the combined effect of the flavonoid and silver nanoparticles could lead to new biomedical applications.

The aim of the present work was the synthesis of Ag_2_O/Ag nanoparticles using Puerarin as reducing and capping agent, followed by the physicochemical characterization and assessment of potential biological activities (e.g., cytotoxicity, potential local irritant and angio-inhibitory effects, antioxidant, and potential antimicrobial activity on pathogenic nosocomial strains) of the formed PUE@Ag_2_O/Ag NPs. Although the syntheses of several Ag NPs obtained using flavonoids were already reported, as well as their biological (particularly antibacterial) and antioxidant properties, we consider that further research is justified, targeting other flavonoids with demonstrated biocompatibility and specific properties. Puerarin was less studied in this respect, and to our best knowledge, this is the first systematic investigation of the synthesis and biological activities of PUE@Ag_2_O/Ag NPs. Another novelty of the present study is the investigation of the in ovo safety profile, targeting the possible utilization of such bioconjugates in cosmetic applications.

## 2. Materials and Methods

### 2.1. Reagents and Microbial Strains

The silver nitrate was purchased from Merck KGaA (54271 Darmstadt, Germany), and Puerarin was obtained from BYOSINTH (CAS [3681-99-0], Bratislava, Slovakia). Our study involved the following reagents and materials: dimethyl sulfoxide ≥ 99.5% (DMSO; Sigma Aldrich, St. Louis, MO, USA), four pathogenic microbial strains (Thermo Scientific, Waltham, MA, USA): *Streptococcus pyogenes* ATCC 19615, *Staphylococcus aureus* ATCC 25923, *Escherichia coli* ATCC 25922, and *Pseudomonas aeruginosa* ATCC 27853; as well as Mueller–Hinton agar (BioMerieux, Marcy-l’Étoile, France) and sheep blood medium cultures (Thermo Scientific, Basingstoke, UK). The blank and antibiotics disks (Gentamicin and Levofloxacin) were purchased from Biomaxima, Poland. Dulbecco’s Modified Eagle Medium (DMEM) was purchased from PAN-Biotech GmbH (Aidenbach, Germany).

### 2.2. Synthesis Route of Ag_2_O/Ag NPs Using Isoflavone Puerarin

For the formulation of Ag_2_O/Ag NPs, the method described in our previous study for the synthesis of ZnO NPs [45] was used with slight modifications. Briefly, 30 mL of silver nitrate solution was heated with a magnetic stirrer at 60 °C, while the 30 mL of preheated isoflavone Puerarin aqueous solution was progressively added until the color of the solution changed from uncolored to dark brown. The reaction was carried out in dark conditions, maintaining the pH at 7.0. The molar ratio between the Puerarin and the precursor was 1:4. After the obtained solution was centrifuged at 10,000 rpm for 15 min, repeated at least three times, then the supernatant was discarded. The precipitate was collected and dried at room temperature for 24 h. The resulting dried product (PUE@Ag_2_O/Ag NPs) was stored in the dark to prevent photo-activation and further characterized. The synthesis was carried out in triplicate, yielding the same UV-Vis absorption spectra and particle size distribution, which confirms the reproducibility of the method.

### 2.3. Characterization Techniques of PUE@Ag_2_O/Ag NPs

Firstly, absorption maxima in the range of 250–800 nm were recorded using UV–Visible spectrophotometry (UviLine 9400 Spectrophotometer, SI Analytics, Deutschland, Germany) to indicate the formation of Ag_2_O NPs. Fourier transform infrared analysis (Bruker Vertex 70 spectrophotometer, Bruker Daltonik GmbH, Bremen, Germany, equipped with a Platinum ATR spectrometer, Bruker Diamond Type A225/Q.I) was used for distinguishing functional groups on the surface of PUE@Ag_2_O/Ag NPs, and 128 scans were recorded in the 4000–400 cm^−1^ range. The X-ray diffraction analysis was carried out using a Rigaku Ultima IV (Rigaku Analytical Devices Inc., Wilmington, MA, USA) X-ray diffractometer, 40 kV, 40 mA with CuKα radiation. The electron microscopy investigations were assessed by TEM (Hitachi HD2700 cold field emission gun STEM microscope equipped with two windowless EDX detectors X-MaxN 100, Chiyoda, Tokyo, Japan) and cold field emission-SEM (Hitachi SU8230 cold field emission gun STEM, Chiyoda, Tokyo, Japan). Dynamic light scattering (DLS, Malvern Zetasizer Nano-ZS device, Malvern PAnalytical Ltd., Malvern, Worcestershire, UK) was utilized to determine the hydrodynamic diameter of PUE@Ag_2_O/Ag NPs, respectively.

### 2.4. Antioxidant Activity Evaluation by DPPH Method

A solution of 2,2-diphenyl-1-picrylhydrazyl (DPPH) was freshly prepared in methanol (0.1 mM), according to the method reported in reference [46], and kept in a dark place at 4 °C until further use. The PUE@Ag_2_O/Ag NPs at varied concentrations (100, 250, 500, 750, and 1000 µg/mL) were determined by DPPH free radical scavenging assay. After 30 min of incubation, the absorbance of each Eppendorf tube sample was determined spectrophotometrically at 517 nm. The sample that contained only DPPH free radical in methanol was used as a blank, while ascorbic acid solution in methanol (tested at the same concentration as the PUE@Ag_2_O/Ag NPs) was used as a positive control. DPPH scavenging activity of the PUE@Ag_2_O/Ag NPs was calculated by the following equation:

$$DPPH\;Scavenging\;Activity\;(\%)=\frac{\left[(A_{DPPH}-A_{PUE@{Ag}_{2}O/Ag\;NPs})\right]}{A_{DPPH}}\times 100$$

where $A_{DPPH}$ is the absorbance of the DPPH free radical solution (blank) without sample, read at 517 nm; $A_{PUE@{Ag}_{2}O/Ag\;NPs}$ is the absorbance of the PUE@Ag_2_O/Ag NPs at various concentrations, in the presence of a 0.1 mM DPPH free radical solution in methanol.

The results obtained were expressed as the IC_50_—meaning the half-maximal inhibitory concentration—calculated by linear regression analysis curve plotting between the DPPH scavenging activity (%) and the concentration of the PUE@Ag_2_O/Ag NPs samples, using OriginLab 2020b software (Origin Lab—Data Analysis and Graphing Software, Szeged, Hungary).

### 2.5. In Vitro Evaluation of the Antibacterial Activity

Antibacterial activity was assessed against four clinically relevant pathogenic bacterial strains, comprising both Gram-positive and Gram-negative species, following the guidelines established by the Clinical and Laboratory Standards Institute (CLSI) recommendations [47]. The tested nosocomial strains included *Streptococcus pyogenes* ATCC 19615, *Staphylococcus aureus* ATCC 25923, *Escherichia coli* ATCC 25922, and *Pseudomonas aeruginosa* ATCC 27853.

#### 2.5.1. Disk Diffusion Method

The disk diffusion method was performed using Mueller–Hinton agar and sheep blood medium cultures to inoculate with bacterial suspensions (0.5 McFarland). Afterward, blank disks were placed on the surface of agar plates and loaded with 15 µL of each sample (500 mg/mL). Disks of Gentamicin served as positive control for pathogenic strains (*Staphylococcus aureus*, *Escherichia coli*, *Pseudomonas aeruginosa*), while Levofloxacin was used for *Streptococcus pyogenes*. The plates were incubated at 37 ± 2 °C for 24 h, after which the diameters of the inhibition zones were measured. The diameter was determined by direct measurement with a calibrated millimeter-scale ruler. Bacterial isolates exhibiting inhibition zones between 7 and 14 mm were classified as resistant to the tested compounds. For strains with inhibition zones greater than 15 mm, MIC testing was conducted.

#### 2.5.2. Dilution Method for Quantifying Minimum Inhibitory Concentration

Using a dilution method, each of the tested samples was diluted in DMSO to obtain decreasing concentrations ranging from 500 to 31.25 mg/mL. In each test tube, 100 µL of each dilution of the test compounds, 50 µL of MH/MHF broth, and 50 µL of the microbial suspension were added. The minimal inhibitory concentration (MIC) was found to be the lowest concentration exhibited by test samples without any visible bacterial growth after 24 h at 37 ± 2 °C.

### 2.6. In Vitro Model–Cell Culturing Protocols

#### 2.6.1. MTT Protocol—Mitochondrial Activity Assessment

The growth of human keratinocytes (HaCaT, CLS 300493, culture cells acquired from CLS—Cell Lines Service GmbH, Eppelheim, Germany) and human malignant melanoma cells (A375, ATCC CRL-1619, culture cells acquired from ATCC, Manassas, VA, USA) in Dulbecco’s Modified Eagle Medium (DMEM) (supplemented with 10% fetal calf serum, 1% L-glutamine, 1% penicillin/streptomycin) was conducted under conditions of 37 °C, 5% CO_2_, and saturated humidity. For these experiments, cells were used while at their exponential phases, 70–80% confluency, and at passages 29–37. Before their use, the materials were dispersed in complete cell media at a 1 mg/mL concentration for 24 h in the incubator at 37 °C. Cells were seeded in 96-well plates at 104 cells/well in 100 µL of complete medium and left to attach and expand for 24 h (until the exponential phase). Cells were in contact with the two samples (Puerarin, PUE@Ag_2_O/Ag NPs) at concentrations ranging from 4 mM to 200 nM. The formed formazan was solubilized with acidified propanol, and its absorbance was read using a spectrophotometer (BioTek Synergy HT plate reader and Gen5 Plate Reader Program) at a wavelength of 550 nm (and 630 nm for background). Each sample underwent 8 replicates (N = 8), with Tween 20 being used as a cell death method control. The untreated control was considered to have 100% viability, and all tested samples were compared to it using the following formula:

$$Mitochondrial\;activity\;(\%)=\frac{{SampleOD}_{550-630}}{{ControlOD}_{550-630}}\times 100$$

#### 2.6.2. Lactate Dehydrogenase (LDH) Released Method—Cytotoxicity Test

After 24 h of contact with the samples in an identical setup, as described above, 50 µL of the cell culture media was transferred to a separate 96-well plate together with 50 µL of phosphate buffer, 50 µL Li-lactate, and 50 µL NAD solution. The mixture was immediately (t_0_) read at the spectrophotometer at 490 nm and 630 nm wavelengths, and then again after 5 min (t_5_). Also, Tween 20 was considered a method control for cell death, and each tested sample had 8 replicates (N = 8). Utilizing the following formula, the LDH release was calculated in mU/mL/min of enzymatic activity.

$$mU/mL/min\;{LDH}_{release}=\left(\frac{\frac{{SampleOD}_{{490-630\;at\;t}_{5}}-{SampleOD}_{{490-630\;at\;t}_{0}}}{5}\times 0.2}{18.4\times 0.625\times 0.05}\right)\times 1000$$

### 2.7. In Ovo Screening Profile Assessment Through HET-CAM Assay

The in ovo experiment (HET-CAM assay) was employed to assess the preliminary biocompatibility profile of the PUE@Ag_2_O/AgO NPs by evaluating their potential to cause local irritation, as well as to investigate their effect on angiogenesis. For this purpose, in ovo experiments were conducted using embryonated chicken eggs prepared according to the methodology described in previous studies [45,48]. To assess local irritation potential, an irritability test was conducted on the ninth day of incubation by applying the test sample to the chorioallantoic membrane (CAM) of embryonated chicken eggs, alongside a positive control (0.5% sodium dodecyl sulfate). A stereomicroscopic evaluation was performed over five minutes to observe potential alterations in the vascular plexus (Sec_Hemorage_, Sec_Lysis_, Sec_Coagulation_). An irritation score was subsequently calculated using the following formula and classified according to Luepke’s criteria as follows: non-irritant (0–0.9), weak irritant (1.0–4.9), moderate irritant (5.0–8.9), and strong irritant (9.0–21.0) [49].

$$Irritation\;score\;\left(IS\right)=\left(5\times \frac{301-{Sec}_{Hemorage}}{300}\right)+\left(7\times \frac{301-{Sec}_{Lysis}}{300}\right)+\left(9\times \frac{301-{Sec}_{Coagulation}}{300}\right)$$

The nanoparticle sample was prepared with 0.5% DMSO to achieve a concentration of 100 µg/mL. Subsequently, 10 µL of the sample was applied within plastic rings positioned over vascularized regions of the chorioallantoic membrane (CAM). Vascular changes induced by the test samples were monitored through live stereomicroscopic analysis and imaging (ZEISS SteREO Discovery.V8, Göttingen, Germany), coupled to a camera (Axiocam 105 color, AxioVision SE64. Rel. 4.9.1 Software). Selected images were further processed using ImageJ open-source software [50]. The experiment was performed in triplicate.

### 2.8. Statistical Analysis

The experimental data obtained in the present study are expressed as mean ± standard deviation. Statistical evaluation of experimental datasets was performed with the help of GraphPad Prism software, version 10.5.0 (GraphPad Software, San Diego, CA, USA). The differences between samples and the control were determined using One-way ANOVA and Dunnett’s multiple comparison tests. The statistically meaningful outcomes were scored using “*”: * *p* ≤ 0.05, *** *p* ≤ 0.001, and **** *p* ≤ 0.0001.

## 3. Results and Discussion

The synthesis of innovative Ag_2_O/Ag NP conjugates was accomplished by using Puerarin, an isoflavone with multiple pharmacological benefits. Nanoparticle formation was initiated by the addition of Puerarin solution (0.025M) to silver nitrate solution (0.1 M) at a molar ratio of 1:4, under continuous stirring at 60 °C and pH = 7. The initial indication of nanoparticle formation was a visible color change in the reaction mixture, followed by the appearance of a precipitate. The formed nanoparticles were isolated by repeated centrifugation and drying at room temperature. As it will result from the following physico-chemical investigations, our study demonstrated that the isoflavone Puerarin mediated the reduction process and the stabilization of the nanoparticles, also attaching itself to the Ag_2_O/Ag NPs. Moreover, the synthesized conjugated nanoparticles exhibited promising biological activities, suggesting their potential utility in medicinal chemistry. Multiple studies have found that silver nanoparticles cause genotoxicity and cytotoxicity in both cancer and normal cell lines, change cell morphology, decrease cell viability in different cell lines by triggering apoptosis through the mitochondrial pathway, and induce ROS generation [51,52,53]. The isoflavone-metal nanoparticle conjugates could modulate these effects, tuning them due to the well-known biological properties of the flavonoid present in the bioconjugate. The investigation of the mechanism and kinetics of Ag_2_O/Ag nanoparticle formation by Puerarin was not the scope of this research, but it can be mentioned that the hypothetical mechanism proposed by several authors and based on the involvement of the -OH groups found in flavonoids in the bioreduction of Ag^+^ [29] could be probably valid for Puerarin, too.

### 3.1. X-Ray Diffraction (XRD) and FTIR Characterization of PUE@Ag_2_O/Ag NPs

**Figure 1:**
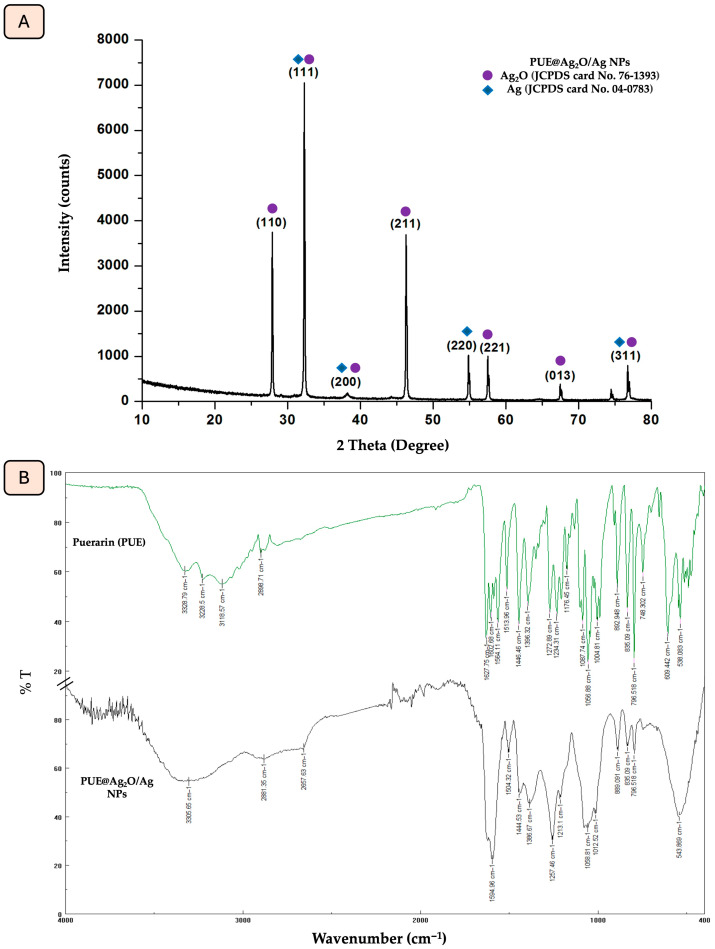
(**A**) X-ray diffraction spectrum of PUE@Ag_2_O/Ag NPs, (**B**) FTIR spectrum of PUE@Ag_2_O/Ag NPs showing distinct peaks, indicating the presence of different functional groups.

Using the X-ray diffraction method, the crystal phases and purity of Ag_2_O/Ag NPs obtained using isoflavone Puerarin are investigated. The XRD spectrum of the synthesized nanoparticles (Figure 1A) shows several sharp and intense Bragg reflection peaks predicted at 2θ degrees at 27.8°, 32.3°, 38.5°, 46.2°, 54.8°, 57.5°, 67.2° and 77.1°, which correspond to the different Miller indices (110), (111), (200), (211), (220), (221), (013) and (311), respectively. These peaks are in agreement with the standard cards (JCPDS card No. 76-1393 and JCPDS card No. 04-0783) and can be attributed to the formation of a face-centered cubic crystal structure [54,55]. The specified diffraction lines can be assigned to the characteristic reflections of the combined phases, including Ag_2_O and Ag.

The identification of functional groups involved in the biosynthesis of PUE@Ag_2_O/Ag NPs was achieved through the ATR-FTIR analysis of both Puerarin and PUE@Ag_2_O/Ag NPs (Figure 1B, Table 1). Puerarin’s dual role as a capping agent was proven through the ATR-FTIR analysis of PUE@Ag_2_O/Ag NPs. In the ATR-FTIR spectrum of PUE@Ag_2_O/Ag NPs, a broad peak of the hydroxyl group was displayed at 3305.65 cm^−1^. The aromatic C-H stretching vibration’s short peak decreased from 2898.71 cm^−1^ to 2881.35 cm^−1^. The carbonyl peak shifted to 1594.96 cm^−1^, and the -C-O vibrational stretching peaks were also moved to 1504.32, 1444.53, and 1058.81 cm^−1^. Silver oxide (Ag_2_O) has distinctive characteristics that occur in the range of 400–1100 cm^−1^. Within this range, a sharp peak appears at 543 cm^−1^, and it was assigned to the Ag-O stretching vibration [56].

### 3.2. UV-Vis Spectroscopy

**Figure 2:**
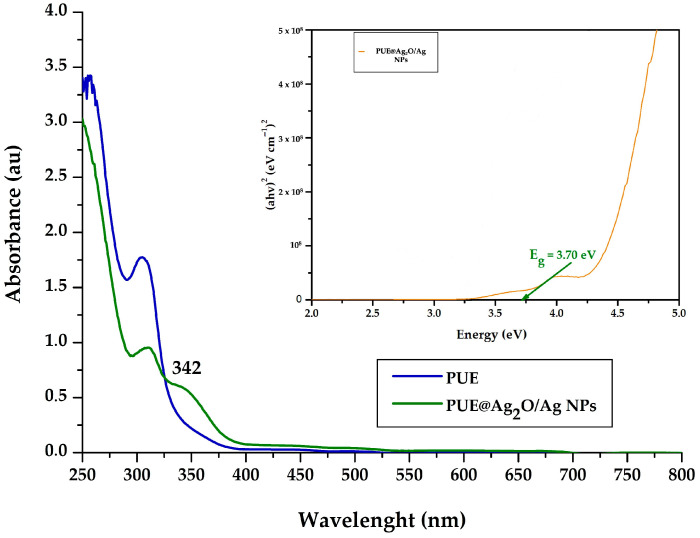
UV-Vis spectrum of Puerarin and PUE@Ag_2_O/Ag NPs with a calculated band gap.

According to the UV-Vis spectra of Puerarin and the PUE@Ag_2_O/Ag NPs (Figure 2), the absorption maximum of the flavonoid and the plasmon resonance maximum of the prepared nanoparticle conjugate were identified at 315 nm and 342 nm, respectively, confirming the bioreduction reaction and the formation of silver oxide nanoparticles as the main components. Although the broad peak, characteristic of most Ag NPs in the 400–500 nm range [57], cannot be observed, it must be specified that shorter wavelengths of the plasmon resonance maxima were also reported for Ag NPs prepared with the flavonoids chrysin (361 nm) or apigenin (346 nm) [58]. This shorter wavelength value also indicates the formation of nanoparticles with a smaller size, but with a tendency towards aggregation, as was confirmed by the DLS measurements (see the results in Section 3.3).

Also, the band gap (Tauc gap) was calculated for all samples using the following formula [59,60], characterized by parameters including the absorption coefficient (α), Planck’s constant (h), frequency (ν), optical band-gap energy (E_g_), and proportionality constant (A).

$${\left(\alpha h\nu \right)}^{2}=A\left(h\nu -E_{g}\right)$$

It is suggested that a narrow band-gap value (e.g., 3.70 eV in our case) will result in more effective photocatalytic activity for future applications as a suitable photocatalytic nanomaterial [9,16].

### 3.3. Particle Size Measurements Through Electron Microscopy and DLS Analysis

**Figure 3:**
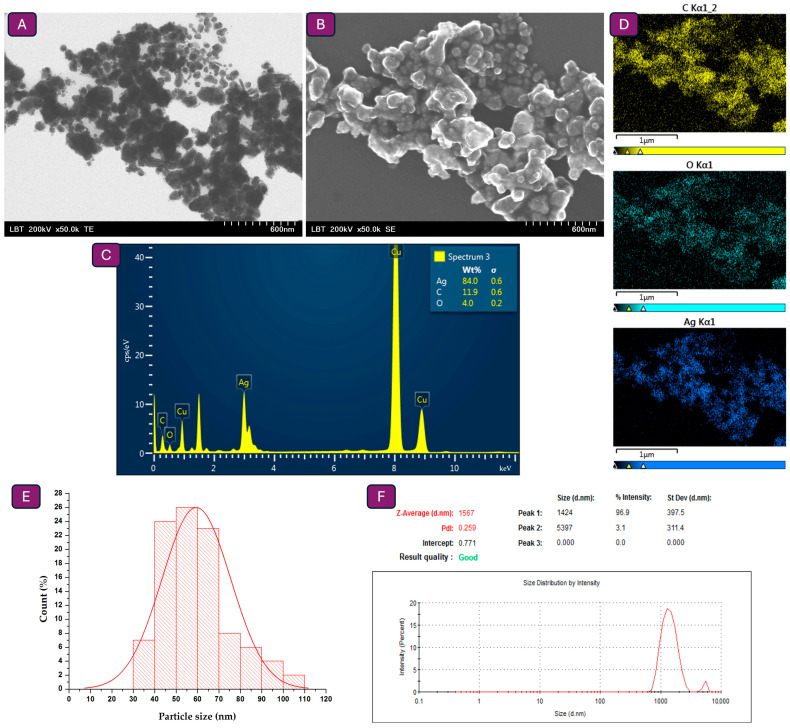
PUE@Ag_2_O/Ag nanoparticles: (**A**) TEM micrographs (×50 k, 200 kV), (**B**) Scanning electron microscopy (SEM) images (×50 k, 200 kV), (**C**) Energy dispersive (EDX) spectrum, (**D**) EDX map, (**E**) Count distribution of particles size, and (**F**) DLS measurements.

TEM analysis (Figure 3A) was employed to evaluate particle size, with measurements performed on a minimum of 100 nanoparticles using ImageJ software, and it was revealed that the nanoparticles are in the nanometric domain (<100 nm) (Figure 3E). On the other hand, according to the SEM analysis, nanoparticles are slightly agglomerated (Figure 3B). The elemental composition of the nanoparticles, evaluated by EDX spectrum and map (Figure 3C,D), confirmed that silver is the predominant element, contributing to more than 70% of the total elemental content, as indicated by its prominent peak. Consequently, the nanoparticle`s surface contains Puerarin, demonstrated by the presence of carbon and oxygen. The DLS analysis (Figure 3F) indicates that the formation of smaller nanoparticles tends to cluster into larger aggregates in suspension (hydrodynamic diameter 1.567 μm, PDI 0.259). According to the literature, PDI values between 0.1 and 0.3 reflect a slightly polydisperse system, characteristic of well-formulated colloidal dispersions, whereas values above 0.5 indicate broad and unstable size distributions [61,62]. After three months of storage, the measurements indicated minimal change, with a hydrodynamic diameter of 1.533 μm and a PDI of 0.251. Hence, the diameter values obtained from the TEM analysis are smaller than those measured by the DLS method, as TEM reflects the physical core size of individual nanoparticles in the dry state, whereas DLS measures the hydrodynamic diameter in suspension, which includes the particle surface coating and potential agglomerates [62,63].

### 3.4. Biological Activities of PUE@Ag_2_O/Ag NPs

The biological activity of the synthesized Ag_2_O/Ag nanoparticle conjugates was investigated to estimate their potential therapeutic applications. Such NPs, particularly when produced through green synthesis using natural compounds and plant extracts, exhibited significant antioxidant, anti-inflammatory, and antimicrobial effects [64,65]. At the same time, numerous studies have shown that Ag NPs have a toxic effect on various cultured mammalian cells. They cause DNA damage, apoptosis, genotoxicity, and chromosome aberrations [66]. We used the MTT assay and the LDH release method to determine the possible modification of this cytotoxic effect by the presence of the isoflavone in the conjugate.

#### 3.4.1. Antibacterial Activity

The potential antibacterial activity of the PUE@Ag_2_O/Ag nanoparticles has been evaluated on *Gram-positive* and *Gram-negative* pathogenic strains. Table 2 presents the results obtained through the disk diffusion and dilution methods. The results demonstrated that PUE@Ag_2_O/Ag NPs exhibited measurable zones of inhibition against all strains tested. The highest inhibition zone (22 mm) was observed against *P. aeruginosa*, followed by *S. aureus* (21 mm), while both *S. pyogenes* and *E. coli* showed inhibition zones of 20 mm. The MIC value for PUE@Ag_2_O/Ag NPs was consistent across all strains at 125 mg/mL, indicating moderate antimicrobial potency.

Despite exhibiting lower antimicrobial potency than the standard antibiotics (e.g., Levofloxacin and Gentamicin), PUE@Ag_2_O/Ag nanoparticles demonstrated broad-spectrum activity against both *Gram-positive* and *Gram-negative* pathogenic nosocomial strains.

Kubavat et al. synthesized Ag NPs through a green approach utilizing rutin and subsequently assessed their antibacterial activity against *Escherichia coli* and *Staphylococcus aureus*. The results revealed that the biosynthesized NPs exerted measurable antibacterial effects on both strains, with the zones of inhibition increasing proportionally to the volume applied. For *E. coli*, inhibition zones of 1.00 mm and 1.75 mm were observed at volumes of 30 µL and 40 µL, respectively. In comparison, *S. aureus* exhibited slightly larger inhibition zones of 1.75 mm and 2.50 mm at the same respective volumes [67]. Another study conducted by Tasca et al. employed quercetin-mediated green synthesis to produce Ag NPs, which were then assessed for their antimicrobial efficacy against *Streptococcus* sp., *Escherichia coli*, and *Candida* sp. Ag NPs with an average diameter of 8 nm exhibited complete inhibition of all tested microorganisms at a minimum inhibitory concentration (MIC) of 1.0 µg/mL, while larger particles (20 nm) required a MIC of 2.5 µg/mL. The pathogenic strain *E. coli* was the most susceptible, with full inhibition observed at just 0.5 µg/mL, using the smaller nanoparticles [68].

#### 3.4.2. Impact on Mitochondrial Activity Using MTT Assay

**Figure 4:**
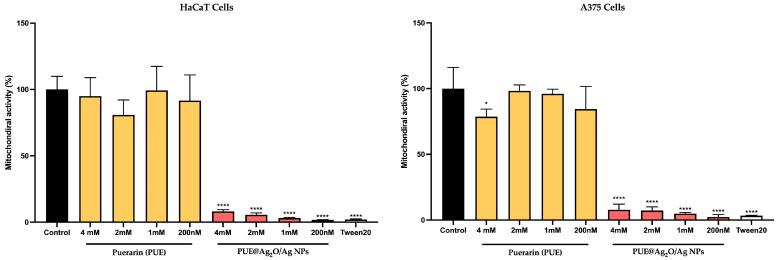
Mitochondrial activity percentage after treatment with PUE@Ag_2_O/Ag nanoparticles on human keratinocytes (HaCaT) and malignant melanoma cells (A375). The statistical analysis performed was a One-way ANOVA comparison post-test (* *p* ≤ 0.05, **** *p* ≤ 0.0001).

According to the MTT cell viability assay, human keratinocytes (HaCaT) and malignant melanoma cells (A375) appear to respond well to the isoflavone Puerarin treatment (Figure 4), as their mitochondrial activity was close to untreated control. Independent of the isoflavone concentration tested (4 mM to 200 nM), the cells’ mitochondrial activity was at 80–100% (±10–20%) activity, which falls within the range of biocompatibility, according to ISO 10993-22 and ISO/TR 10993-22 [69]. The previous literature reports that silver nanoparticles exhibit dose-dependent cytotoxic effects, with values typically ranging from 1 to 100 µg/mL (approximately 0.01–1 mM), depending on the size, surface functionalization, and cell type [70,71,72]. In comparison, the PUE@Ag_2_O/Ag NPs were cytotoxic for both cell types at all tested concentrations, showing mitochondrial activities below 10%. Gross cell death has been observed in this response, which is very similar to the one observed in the control sample (Tween20).

#### 3.4.3. Cytotoxicity Evaluation via the LDH Release Method

**Figure 5:**
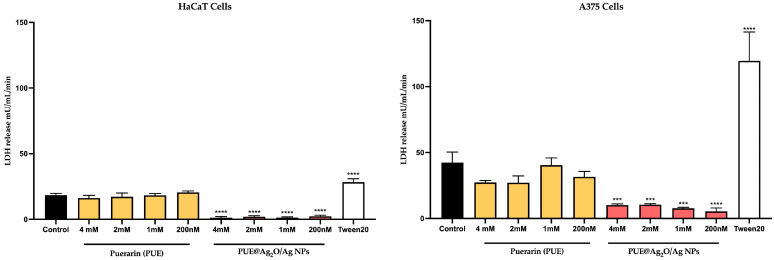
Cytotoxicity percentage after treatment at four different concentrations (1 mM, 2 mM, 4 mM, 200 nM) for an interval of 24 h, on human keratinocytes (HaCaT) and malignant melanoma cells (A375). The statistical analysis performed was a One-way ANOVA comparison post-test (*** *p* ≤ 0.001, **** *p* ≤ 0.0001).

The LDH assay revealed that untreated (control) keratinocytes and melanoma cells release lactate dehydrogenase (18.45 ± 1.3 mU/mL—HaCaT; 42.47 ± 7.9 mU/mL—A375) in the medium (Figure 5), probably as a result of extracellular vesicle release [72]. In comparison, cells treated with isoflavone Puerarin presented similar LDH values, slightly decreasing with the increase in concentration for HaCaT, but with a statistically significant decrease for melanoma cells (27–40 mU/mL) at three of the four tested concentrations. This decrease could be indicative of reduced cell population or reduced export of vesicular content when compared to the control. The PUE@Ag_2_O/Ag nanoparticles presented very low LDH release for both cell types (<5 mU/mL—HaCaT, <14 mU/mL—A375), compared to the untreated control, which could indicate a general lack of cells in the population (if considering the MTT results as well).

#### 3.4.4. Antioxidant Activity Using DPPH Assay

**Figure 6:**
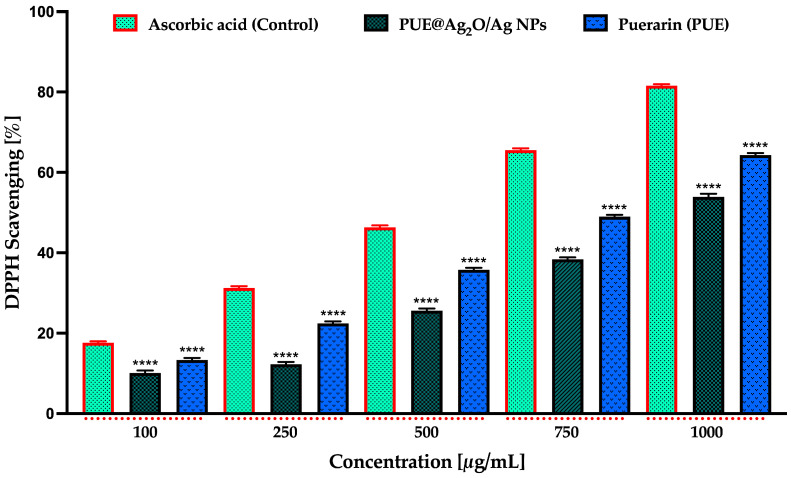
Bar graph of percent DPPH radical scavenging activity for ascorbic acid (control), PUE@Ag_2_O/Ag NPs, and isoflavone Puerarin (PUE). Data are presented as mean ± SD (*n* = 3). One-way ANOVA followed by Dunnett’s multiple comparisons test was performed for quantifying graphically represented data (**** *p* ≤ 0.0001).

Different concentrations of PUE@Ag_2_O/Ag nanoparticles (100, 250, 500, 750, and 1000 μg/mL) were evaluated for their antioxidant activity. The antioxidant activity percentage obtained for all the concentrations tested of the PUE@Ag_2_O/Ag NPs sample, as well as for the isoflavone and the ascorbic acid samples, represents an average of three measurements ± standard deviation (SD). As illustrated in Figure 6, the antioxidant effect of the nanoparticles was dose-dependent, with the highest concentration (1000 μg/mL) exhibiting the greatest radical scavenging activity. However, this activity remained lower than that of the standard control, ascorbic acid, and of the isoflavone Puerarin. At 1000 μg/mL, PUE@Ag_2_O/Ag NPs, ascorbic acid, and Puerarin demonstrated maximum DPPH radical scavenging activities of 53.79 ± 0.38%, 81.77 ± 0.55%, and 64.36 ± 0.47%, respectively, (Figure 6).

Further, by linear regression analysis, the IC_50_ was calculated between the antioxidant activity values and their related concentrations. The IC_50_ of PUE@Ag_2_O/Ag NPs was 981.5 ± 94.2 μg/mL (R^2^ = 0.95811), IC_50_ = 713.9 ± 65.3 μg/mL of Puerarin (R^2^ = 0.96141) and the IC_50_ of the control (methanolic solution of ascorbic acid) was 452.6 ± 49.9 μg/mL (R^2^ = 0.93429).

Ag_2_O/Ag nanoparticles, particularly when produced through green synthesis using natural compounds and plant extracts, exhibit significant antioxidant and antimicrobial effects [65,73]. In a study conducted by Kubavat et al., silver nanoparticles synthesized using rutin exhibited strong antioxidant potential, with DPPH radical scavenging activity reaching 86.95 ± 1.60% at a concentration of 80 μg/mL [67]. Another example, in a study conducted by Chahardoli et al., quercetin-assisted silver nanoparticles demonstrated dose-dependent antioxidant activity, achieving 82.3% DPPH radical scavenging at 400 μg/mL. Additionally, the nanoparticles exhibited 47% hydrogen peroxide scavenging at the same concentration, surpassing the activity of ascorbic acid [74].

To place our findings in a broader context, we conducted a comparative analysis with other green-synthesized silver nanoparticles using flavonoids (Table 3).

In accordance with the comparative data presented in Table 1, our PUE@Ag_2_O/Ag NPs differ from other green-synthesized Ag NPs in terms of physicochemical properties while still maintaining dimensions consistent with the expected nanoscale range. From a biological perspective, the nanoparticles demonstrated moderate antibacterial activity against both Gram-positive and Gram-negative strains, with MIC values comparable to those reported for quercetin-based Ag NPs. Antioxidant activity, assessed by IC_50_, suggests that Puerarin contributes less effectively to radical scavenging in nanoparticle form, especially when compared to quercetin, rutin, or myricetin-derived Ag NPs. Notably, cytotoxicity analysis revealed a pronounced effect: while Ag NPs based on apigenin or rutin exhibited dose-dependent or minimal cytotoxicity, PUE@Ag_2_O/Ag NPs induced strong mitochondrial inhibition at all tested concentrations, with minimal LDH release. This profile suggests the possible utility of these nanoparticles in targeted anti-cancer or localized antimicrobial applications.

#### 3.4.5. Irritant Potential and Influence of PUE@Ag_2_O/Ag NPs on In Ovo Angiogenesis

**Figure 7:**
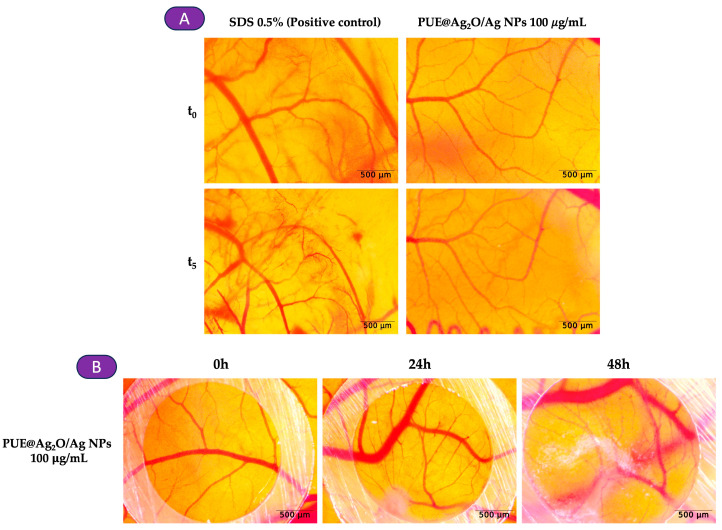
Representative images of PUE@Ag_2_O/Ag nanoparticles evaluated using the HET-CAM assay: (**A**) Stereomicroscopic images of the chorioallantoic membrane after treatment with SDS 0.5% (positive control), and PUE@Ag_2_O/Ag NPs at a concentration of 100 μg/mL; (**B**) Angiogenesis assessment of PUE@Ag_2_O/Ag NPs, stereomicroscope images represent the 24 h, respectively, 48 h modification upon the treated vascular plexus; scale bars represent 500 µm.

Using the HET-CAM assay, the irritation score was calculated as the outcome of our investigation of the potential local irritant and angio-inhibitory effects of PUE@Ag_2_O/Ag (Figure 7, Table 4). The PUE@Ag_2_O/Ag NPs were classified as non-irritating because no signs of irritation appeared during the 5 min stereomicroscopic evaluation. However, in the case of the SDS 0.5% (positive control), which caused vascular events and was thus classified as a severe irritant substance (Figure 7A), we applied a similar approach to the previous assay and monitored stereomicroscopically to determine if any angio-inhibitory effects could be detected. The nanoparticles exhibit good vascular tolerance, with no significant changes or anomalies observed in the normal developing vascular plexus after 24 h and 48 h post-treatment, which suggests that the angiogenesis process was not affected (Figure 7B).

## 4. Conclusions

The Ag_2_O/Ag nanoparticles were successfully synthesized using the isoflavone Puerarin as a green reducing and capping agent. XRD and FTIR analyses confirmed the formation of face-centered cubic Ag_2_O/Ag nanoparticles and the presence of Puerarin on their surface. TEM and SEM analyses revealed quasi-spherical and hexagonal nanoparticles in the nanometric domain (<100 nm).

Ag_2_O/Ag nanoparticles exhibited high toxicity to both cell lines at all tested concentrations, causing a significant reduction in mitochondrial activity and cell death. PUE@Ag_2_O/Ag nanoparticles (100 μg/mL) were classified as non-irritating in the HET-CAM assay. The nanocomposite showed good vascular tolerance and did not significantly affect the angiogenesis process in the chorioallantoic membrane model. Additionally, the nanoparticles exhibit promising potential as antimicrobial agents, supported by their antioxidant capacity and effective antimicrobial activity against key pathogenic nosocomial strains (*Streptococcus pyogenes*, *Staphylococcus aureus*, *Escherichia coli*, *Pseudomonas aeruginosa*).

Overall, the green synthesis method using Puerarin successfully produced Ag_2_O/Ag nanoparticles with controllable properties. While the nanoparticles showed promising characteristics in terms of synthesis and physical properties, their high cytotoxicity limits their potential for biomedical applications. The in ovo results suggest that the nanoparticles may have greater compatibility in certain biological systems compared to in vitro cell cultures. While the nanoparticles exhibited cytotoxicity in vitro, the absence of irritation and angiogenesis inhibition in the in ovo HET-CAM model suggests a more complex biocompatibility profile.

Finally, the conclusions highlight the successful synthesis of Ag_2_O/Ag nanoparticles using a green method but also emphasize the need for further research to address cytotoxicity issues before considering biomedical applications. Further in-depth experimental studies are required to improve biocompatibility and comprehensively evaluate the cytotoxicity profiles of these conjugates to ensure their safety for therapeutic use. Various methods, particularly particle size and surface modification, will be investigated to optimize the loading of the Ag_2_O/Ag nanoparticles with Puerarin, in order to decrease the cytotoxicity. Future studies will also explore mechanistic pathways (e.g., ROS generation, apoptosis induction) and extend evaluations to in vivo models for comprehensive safety and efficacy validation.

## Figures and Tables

**Table 1:** FT-IR spectra absorption peaks with potential assignments for both PUE@Ag_2_O/Ag NPs and isoflavone Puerarin (PUE).

Possible Assignment for Compound Class	Absorption Peaks of Puerarin (cm^−1^)	Absorption Peaks of PUE@Ag_2_O/Ag NPs (cm^−1^)
O-H stretching vibrations	3328.79 3228.5	3305.65
C-H aromatic vibration	3118.57 2898.71	2881.35
C=O stretching vibrations of the aryl ketone group	1627.75	1594.66
-C-O vibrational stretching	1564.11 1513.96 1446.46 1056.88	1504.32 1444.53 1058.81
Ag-O stretching vibration	-	543.86

**Table 2:** Antimicrobial activity of standard antibiotics and PUE@Ag_2_O/Ag nanoparticles.

Pathogenic Strains	Test Sample	Disk Diffusion Method (Inhibition Zones in mm)	Minimum Inhibitory Concentration (mg/mL)
*Streptococcus pyogenes* ATCC 19615	PUE@Ag_2_O/Ag NPs Levofloxacin 5 µg	20 24	125 * NA
*Staphylococcus aureus* ATCC 25923	PUE@Ag_2_O/Ag NPs Gentamicin 10 µg	21 26	125 * NA
*Escherichia coli* ATCC 25922	PUE@Ag_2_O/Ag NPs Gentamicin 10 µg	20 26	125 * NA
*Pseudomonas aeruginosa* ATCC 27853	PUE@Ag_2_O/Ag NPs Gentamicin 10 µg	22 23	125 * NA

* NA—no activity.

**Table 3:** Comparative characteristics of green-synthesized silver nanoparticles using various flavonoids.

Polyphenolic Compound	Physico-Chemical Parameters, Antioxidant and Antimicrobial Activities, Cytotoxicity Results	References
**Quercetin**	spherical, smooth surface zeta potential = −15.1 + 3.60 mV hydrodynamic particle size = 310.4 nm *Escherichia coli*: sample 1 (containing water and silver nanoparticles): zone of inhibition = 4.2 cm; and sample 2 (containing silver nanoparticles in methanol): zone of inhibition = 4.4 cm.	[75]
	UV-Vis intense absorption peak at 420 nm according to TEM and SEM analyses, the nanoparticles exhibited a size = 30 nm antioxidant dose-dependent nature, at 80 μg/mL = 69%.	[76]
	UV-Vis absorption peak of Ag NPs at 413.42 nm	[77]
	UV-Vis absorption peak at 391 nm average size in the range of 20 ÷ 40 nm antioxidant activity IC_50_ = 100.4 µg/mL	[78]
	UV-Vis absorption peak at 391 nm hydrodynamic nanometer size = 86.6 PDI = 0.18 ± 0.04 *S. aureus*: MIC = 62.5 µg/mL, MBC = 250 µg/mL E. coli: MIC = 125 µg/mL, MBC = 500 µg/mL	[79]
**Apigenin**	hydrodynamic diameter = 35.6 nm diameter size = 13.9 ± 5.6 nm (according to TEM analysis) antioxidant properties at lower concentrations and pro-oxidant properties at higher concentrations low toxicity observed at ≤4 μg/mL; significant toxicity detected at ≥8 μg/mL, though nanoparticles remain less toxic even at 256 μg/mL	[37]
**Rutin**	UV-Vis absorption peak at 273 nm mean hydrodynamic diameter = 115 nm PDI = 0.181 average size = 14.5 ± 1.8 nm (according to TEM analysis) HUVEC (human umbilical vein endothelial cells) viability gradually decreased with increasing AgNP concentration but remained above 80% at concentrations up to 100 mg/L	[80]
	UV-Vis absorption peak at 425 nm average size = 80–85 nm zeta potential = −30.3 mV DPPH method: 86.95 ± 01.60% Antimicrobial activity on *Escherichia coli* (1.75 mm inhibition zone), and *Staphylococcus aureus* (250 mm inhibition zone)	[67]
**Myricetin**	UV–Vis absorption at 410 nm DPPH method: free radical-scavenging rate = 60–87% *Escherichia coli* (MIC = 10^−4^ g/L) and *Salmonella* (MIC = 10^−5^ g/L)	[38]
**Gallic acid**	UV–Vis absorption at 410 nm nanoparticle diameter size = 15 nm	[81]
	UV–Vis absorption at 400 nm hydrodynamic diameter = 17.6 ± 5.3 nm *E. coli*—MIC = 6 μg/mL, *S. aureus*—MIC = 30 μg/mL, *C. albicans*—MIC = 24 μg/mL nanoparticles showed dose-dependent cytotoxicity, with higher concentrations (>24 μg/mL) being more toxic to HeLa cancer cells than to HL-7702 normal cells.	[82]
**Puerarin**	UV-Vis absorption at 342 nm hydrodynamic diameter = 1.567 μm PDI = 0.259 band-gap value = 3.70 eV antimicrobial activity: *P. aeruginosa* (inhibition zone 22 mm), *S. aureus* (21 mm), *S. pyogenes,* and *E. coli* (20 mm). The MIC value was consistent across all strains at 125 mg/mL, indicating moderate antimicrobial potency. antioxidant activity: dose-dependency, IC_50_ = 981.5 ± 94.2 μg/mL LDH release for both cell types (<5 mU/mL—HaCaT, <14 mU/mL—A375) cytotoxic for both cell types at all tested concentrations, showing mitochondrial activities below 10% in ovo studies (HET-CAM Assay): non-irritating and exhibit good vascular tolerance, with no significant changes or anomalies	Our study

**Table 4:** Irritability evaluation using the HET-CAM assay for PUE@Ag_2_O/Ag NPs (in concentration of 100 μg/mL) and positive control samples (SDS 0.5%).

Sample	Irritation Score (IS) *	Irritation Category/Type of Effect
**Positive control—SDS 0.5%**	15.58 ± 0.24	Strongly irritant
**PUE@Ag_2_O/Ag NPs, 100 μg/mL**	0 ± 0	Non-irritant

* In accordance with the Luepke scale [49]. One-way ANOVA post-test was used for comparison among groups (*p* < 0.001 versus control).

## Data Availability

The original contributions presented in the study are included in the article, further inquiries can be directed to the corresponding author.
